# Personal Listening device (PLD) usage among University Students and their audiometric profile during the shift to online learning post COVID-19

**DOI:** 10.1371/journal.pone.0319665

**Published:** 2025-03-25

**Authors:** Ana’am Alkharabsheh, Sara Alshurafa, Sara Alhanbali, Soha Garadat

**Affiliations:** 1 Department of Hearing and Speech Sciences, Faculty of Rehabilitation Sciences, University of Jordan, Amman, Jordan; 2 Department of Audiology and Speech-Language Pathology, A.T. Still University-Arizona School of Health Sciences, Mesa, Arizona, United States of America; Universiti Malaya Fakulti Perubatan: University of Malaya Faculty of Medicine, MALAYSIA

## Abstract

As the online learning increases post COVID-19 and in corresponding to the rise in the personal listening devices use, the present study aims to describe personal listening device (PLD) usage and auditory profile among a nationally sample of university students in Jordan and examine audiometric outcomes among them. The relationship between the usage of personal listening devices and hearing acuity will be examined. The relation between hours of personal listening devices use, volume levels, and self-report measures will additionally be explored. A total of 65 students from the University of Jordan were recruited. A full audiological tests battery including; routine puretone audiometry, extended high-frequency audiometry, and distortion product otoacoustic emissions, was conducted. Also, students were asked to fill the Arabic version of Tinnitus Handicap Inventory (THI) questionnaire and Hyperacusis Questionnaire (HQ). The use of personal listening devices does not seem to be related to the hearing threshold in the standard frequency range of 0.25-8 kHz for the majority of participants in this study. Using high-frequency audiometry, current results indicated a significant association between the usage of personal listening device and hearing thresholds at 18 and 20 kHz. While the distortion product otoacoustic emissions results showed that the signal to noise ratio (SNR) was affected (SNR < 6 dB) in only 20% of the students. With online learning becoming widely accepted as a standard educational approach post COVID-19, there is an anticipated surge in the usage of personal listening devices. Understanding the potential impact of this transition on the auditory well-being of students is crucial for ensuring their overall health and academic success. The use of personal listening devices for online learning might introduce potential risks to hearing and could align with the unsafe listening practices observed among young adults.

## Introduction

The COVID-19 pandemic, declared by the World Health Organization (WHO) as a global health crisis on March 11th, 2020, significantly disrupted daily life worldwide. This disease, caused by Severe Acute Respiratory Syndrome coronavirus 2 (SARS-CoV-2), led to millions of deaths globally and prompted widespread changes across multiple sectors, including education [1,2]. To mitigate the spread of the virus, educational institutions rapidly transitioned to online learning platforms, a shift that affected approximately 1.725 billion learners across 200 countries by July 2020 [3–6].

Online learning relies heavily on technology, including personal listening devices (PLDs) such as earbuds and headphones, which have become indispensable tools for many students. While PLDs provide significant benefits, including accessibility and convenience, their excessive use has raised concerns regarding auditory health. Previous studies indicate that up to 90% of teenagers and young adults use PLDs regularly, often at unsafe volume levels [7–9]. The World Health Organization (WHO) has highlighted the risks of hearing loss associated with such practices, noting that hazardous sound levels can exceed 100 dBA, with some devices capable of reaching levels as high as 128 dBA [10–13]. Moreover, in noisy environments, users frequently increase the volume to counteract background noise, further exacerbating the risk of hearing damage [14].

Research has shown that prolonged exposure to loud noise, defined as levels exceeding 85–89 dB for more than eight hours daily, can cause permanent hearing damage, commonly referred to as noise-induced hearing loss (NIHL) [15–22]. Such exposure can lead to temporary threshold shifts (TTS), which, if repeated, may result in irreversible damage to the cochlea and auditory system. This damage is associated with auditory disorders such as tinnitus—a perception of ringing or buzzing in the absence of external stimuli—and hyperacusis, an increased sensitivity to normal environmental sounds [23–31]. These conditions can profoundly impact quality of life and underscore the need for preventive measures.

The shift to online learning during the COVID-19 pandemic has likely increased students’ reliance on PLDs, with daily screen time rising from an average of five hours’ pre-pandemic to six, eight, or even ten hours during lockdowns [32–34]. Such prolonged usage may amplify the risk of auditory health issues. Despite these concerns, no prior studies have specifically investigated the impact of PLD use during online learning on students’ hearing health.

This study aims to address this critical gap by examining the patterns of PLD usage for online learning and their impact on auditory health among a nationally representative sample of university students in Jordan. Specifically, the study seeks to evaluate the prevalence of PLD use, assess hearing profiles through audiometric measures, and identify associations between prolonged PLD use and auditory disorders such as noise-induced hearing loss, tinnitus, and hyperacusis. By filling this gap, the study aims to provide evidence-based insights into the risks posed by increased PLD use during the pandemic and contribute to the development of targeted preventive strategies.

## Materials and methods

### Participants

A total of 65 students, aged 19-24 years (60 females, 5 males) and representing all academic levels from the university campus, were recruited via social media platforms (student groups) and university email lists between March 15, 2023, and June 30, 2023. A convenience sampling method was used, with the sample size determined by participant availability. Recruitment advertisements emphasized the voluntary nature of participation and included detailed information about the study objectives.

Inclusion criteria were students who used PLDs bilaterally and were actively engaged in online learning during the study period. Exclusion criteria included individuals with ear infections, middle ear conditions, or those taking medications known to affect auditory performance. Acoustic immittance measurements were conducted on all participants to rule out middle ear dysfunction; the Otometrics Madsen Otoflex 100 tympanometer was used for this purpose. Participants with abnormal results were excluded from further testing. None of the participants reported any illness on the day of testing. All participants provided written informed consent and were informed of their option to withdraw from the study at any time. This study was reviewed and approved by the Deanship of Scientific Research at the University of Jordan (2022-23/IRB) (Supporting Information 1).

### Assessment measures and procedures

Data collection involved administering a detailed, electronically distributed questionnaire and conducting comprehensive auditory assessments. The questionnaire, developed based on prior validated tools and expert consultation, included sections on demographic information, hearing history (including the presence and duration of tinnitus and/or hyperacusis), and PLD usage patterns. Questions covered the type of PLDs used, duration of daily use, and purpose of use (educational or recreational). To ensure clarity and reliability, the questionnaire was piloted with a sample of 10 students, and necessary adjustments were made before full deployment.

Hearing status was assessed using both behavioural and objective measures. Pure-tone audiometry was conducted to assess thresholds in the standard frequency range of 0.25-8 kHz using TDH-39 headphones. Extended high-frequency audiometry (10-20 kHz) was conducted using Sennheiser HDA 200 circumaural headphones. Both assessments were performed in a sound-treated booth using a clinical two-channel audiometer (Interacoustics, AC40, Denmark), calibrated to ANSI S3.6-1996 standards [35]. Thresholds were obtained for both ears and recorded for analysis.

Objective auditory assessments included distortion product otoacoustic emissions (DPOAE) testing, which evaluates outer hair cell integrity and cochlear function. Measurements were conducted using the Otometrics Madsen Capella device, with primary tones f1 and f2 set at a ratio of 1.22. Frequencies ranging from 1-10 kHz were tested, and DPOAEs were considered present if the signal-to-noise ratio (SNR) was ≥  6 dB. These assessments were conducted by trained audiologists to ensure accuracy and consistency.

Tinnitus and hyperacusis were evaluated using the translated versions of the Tinnitus Handicap Inventory (THI) and Hyperacusis Questionnaire (HQ) [36,37]. The THI, consisting of 25 items, assessed the impact of tinnitus on daily life, with responses scored as Yes (4 points), Sometimes (2 points), or No (0 points). An average score was calculated for each participant. The HQ, a 20-item questionnaire, evaluated hypersensitivity to sounds, with higher scores indicating greater sensitivity or intolerance. Both questionnaires were administered only to students reporting tinnitus and/or hyperacusis. These tools have been validated in previous studies, ensuring their suitability for assessing the target conditions.

### Statistical analysis

Data were analysed using the Statistical Package for Social Sciences (version 22; SPSS Inc., Chicago, IL, USA). Normality of the data was checked using Shapiro-Wilk and Kolmogorov-Smirnov tests. Kruskal Wallis test was used to identify the presence of significant difference of DPOEAs results (SNRs) and the hearing thresholds at the different frequencies tested between the two ears.

For the THI and HQ questionnaires, the score means and standard deviations were calculated. Crosstabs was used to find the association between self-report of tinnitus, hyperacusis and the hours of usage and the volume level. Descriptive analysis was reported using frequency distribution and percentage for the study variables including prevalence of hearing loss, tinnitus, hyperacusis, and tests results; including routine pure tone audiometry and extended high frequencies, and otoacoustic emissions. Interaction effects between variables such as PLD usage and hearing outcomes were examined through these statistical methods.

## Results

### Independent and dependent variables

Independent Variables: Type of headphones (headset vs. over-ear), usage duration (hours per day), and volume level (moderate, high, very high) of personal listening devices (PLDs), as well as participation in online learning.

Dependent Variables: Hearing sensitivity (as measured by Puretone Audiometry (PTA) and Distortion Product Otoacoustic Emissions (DPOAEs)), self-reported tinnitus and hyperacusis symptoms, and the association between these variables and PLD usage patterns.

### Puretone Audiometry (PTA)

**Fig 1:**
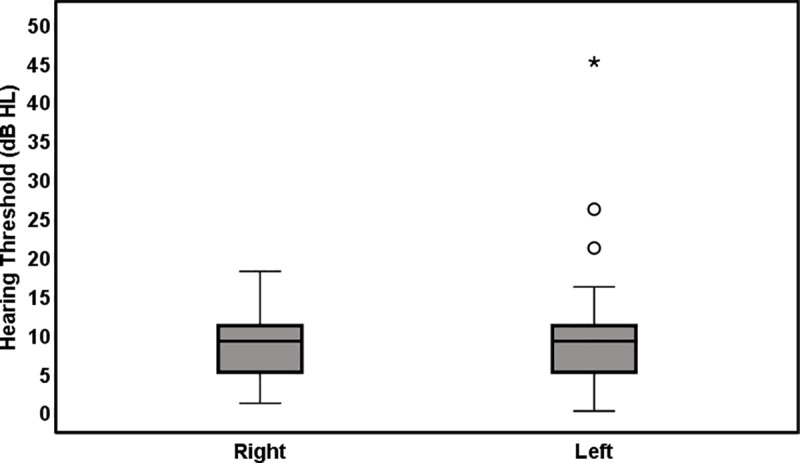
The box plot represents the PTA results for the right and left ears. The central line in each box indicates the median, and the whiskers represent the data spread. Outliers are marked as dots. PTA >  25 dB HL, indicating abnormal hearing, was observed in three students, all in the left ear.

Fig 1 shows the Puretone Audiometry (PTA) results at 0.5, 1, and 2 kHz, reflecting hearing sensitivity in the speech frequency range. The average PTA for the right ear was 8.70 ±  3.83 dB HL, and for the left ear, it was 9.09 ±  6.39 dB HL, indicating normal hearing sensitivity for most participants. Audiometric testing across the standard frequency range (0.25-8 kHz) confirmed normal hearing sensitivity for the majority of participants. Only three participants exhibited hearing loss, with PTA values greater than 25 dB HL.

The Kruskal-Wallis test was used to compare hearing thresholds between the right and left ears within the extended high-frequency range (10-20 kHz). The results showed no significant differences between the ears (p >  0.05), allowing for data pooling. Based on established norms [38], no significant hearing loss was found in the 10-16 kHz range. However, 50% of students showed abnormal hearing at 18 kHz, and 95% exhibited abnormal hearing at 20 kHz.

### Distortion Product Otoacoustic Emissions (DPOAEs)

**Fig 2:**
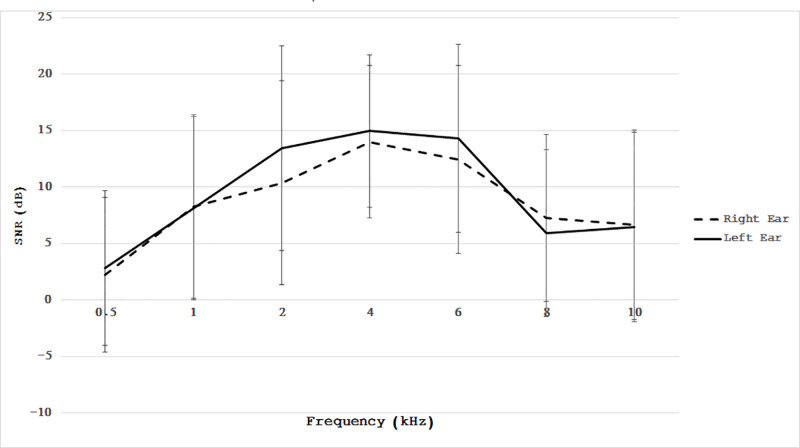
The line graph illustrates the SNR levels for DPOAEs in the left and right ears across frequencies from 1 to 10 kHz. No significant differences in DPOAE outcomes were found between ears across this range.

Fig 2 shows the DPOAE results across the frequency range of 1-10 kHz for both ears. The Kruskal-Wallis test revealed no significant differences in signal-to-noise ratios (SNR) between the right and left ears (p >  0.05). Of the 65 subjects, 20% exhibited abnormal DPOAEs (SNR <  6 dB) in both ears.

### Self-reported measures

Regarding self-reported hearing loss, three students indicated hearing loss through a binary question (“Do you have hearing loss?”). Tinnitus was reported by 22 students (34%), with 14 of these having tinnitus scores ≥ 24 (average score: 24.76 ±  20.54). The distribution of tinnitus severity was as follows: 7 students reported no tinnitus (score: 0-16), 8 students had mild tinnitus (score: 18-36), 4 had moderate tinnitus (score: 38-56), 1 had severe tinnitus (score: 58-76), and 1 had bothersome tinnitus (score: 78-100). One student did not complete the tinnitus questionnaire.

Regarding hyperacusis, 18 students (27.7%) reported symptoms (mean score: 43.20 ±  19.23). A score greater than 28 on the hyperacusis questionnaire suggests a strong sensitivity to sound.

The type of headphones used by participants was primarily headsets (87.7%), with 12.3% using over-ear headphones. There was no significant association between headphone type and self-reported tinnitus (χ² (1) =  0.32, p >  0.05) or hyperacusis (χ² (1) =  1.05, p >  0.05). However, a higher proportion of students with tinnitus (20 out of 22) and hyperacusis (17 out of 18) used headsets, suggesting a potential association with the use of personal listening devices.

### Hours of usage

The average duration of PLD use for leisure was 3.10 ±  4.71 hours, and for online learning, it was 5.09 ±  6.19 hours. Online learning contributed an additional 5 hours of PLD use, bringing the total to 8 hours. However, no significant associations were found between the hours of PLD use and the self-reported occurrence of tinnitus (χ² (22) =  16.66, p >  0.05) or hyperacusis (χ² (22) =  19.89, p >  0.05).

### Volume level

Regarding the intensity of PLD use, 50% of students reported using moderate volume, while 41.5% used high volume and 12.3% used very high volume. No significant association was found between the volume level and self-reported tinnitus (χ² (2) =  1, p >  0.05) or hyperacusis (χ² (2) =  4.10, p >  0.05).

## Discussion

### Association between the use of personal listening devices and hearing thresholds

The association between the usage of PLDs and hearing thresholds has been documented across numerous studies, yet the specific association between the increased PLD use for online learning during the COVID-19 pandemic and hearing health has not been extensively explored. The shift to online learning was necessitated by safety measures, creating an environment where PLDs were commonly used, which continued into post-pandemic education. The widespread use of PLDs during this period raises important questions regarding potential effects on auditory health, warranting further examination.

This study aimed to describe PLD usage and auditory profile among a nationally representative sample of university students in Jordan and examine audiometric outcomes among them. Results indicate that, for most participants, the use of PLDs did not significantly affect hearing thresholds within the standard frequency range of 0.25–8 kHz. However, three students reported elevated thresholds (>25 dB), in line with the American Speech-Language-Hearing Association’s criteria [39]. These results are consistent with previous research suggesting that pure-tone audiometry (PTA) might not fully capture cochlear damage that may occur from prolonged high-volume exposure [31,40,41]. For example, Pawlaczyk-Łuszczyńska et al. [42] reported that most students who used portable audio devices had normal hearing, likely because NIHL generally develops gradually over extended exposure periods. The ISO 1999 model [43], which posits that exposure to noise levels of 85 dB(A) for 15 years or 90 dB(A) for six years can result in hearing threshold shifts, supports the concept that more extended PLD exposure may be necessary to observe discernable threshold shifts [44,45].

High-frequency audiometry results in this study suggest that hearing thresholds at 18 and 20 kHz might be impacted, representing the upper limit of human auditory perception. However, the clinical relevance of high-frequency threshold changes remains uncertain, as it is not fully understood if these observations indicate genuine auditory damage or are due to equipment limitations. Thus, these findings should be interpreted cautiously and considered preliminary rather than conclusive. Further diagnostic tests such as speech-in-noise assessments and audiograms beyond PTA may be beneficial to capture more nuanced hearing health indicators [44,45]. Additionally, variables like PLD volume levels and usage patterns play a role in potential hearing outcomes, although no significant threshold shifts were evident in this study at the time of testing.

Regarding distortion product otoacoustic emissions (DPOAEs), the study found that approximately 20% of participants exhibited signal-to-noise ratios (SNRs) below 6 dB. While this aligns with findings from Le Prell et al. [46], who did not associate common recreational sound exposure with DPOAE amplitude, this suggests that some students may have experienced preclinical auditory damage due to high-intensity PLD use. Longitudinal studies are needed to ascertain the possible long-term risks associated with such usage patterns.

### The effect of PLDs on tinnitus and hyperacusis

Although no apparent impact on hearing thresholds was observed, 21.5% of participants reported tinnitus based on Tinnitus Handicap Inventory (THI) scores, indicating the presence of tinnitus despite normal audiometric thresholds. Research suggests that tinnitus may occur in individuals with normal hearing sensitivity due to synaptic loss between inner hair cells and auditory nerve fibers following excessive noise exposure. Interestingly, this synaptic loss remains undetected through standard audiograms, but it can lead to alterations in the neural pathways within the auditory system [47]. Additionally, current findings indicate a connection between THI and HQ scores and self-reports of tinnitus and hyperacusis. We found that 14 out of 22 students who completed the THI questionnaire had scores from mild to severe tinnitus, with a cutoff score of 24 (the cutoff point for THI score was 24) [48–50]. Similarly, 8 out of 18 students who completed the Hyperacusis Questionnaire (HQ) had scores ≥ 28, which is the designated threshold for identifying hyperacusis [51]. Surprisingly, no significant correlation emerged between PLD volume, usage duration, and tinnitus or hyperacusis outcomes, suggesting online learning during the pandemic could contribute to auditory challenges such as tinnitus, although causation cannot be assumed based on current data.

The availability of PLDs and lack of stringent regulation likely contributed to the widespread practice of unsafe listening habits among young people [52]. Given that students also engaged in recreational listening, evaluating the specific impact of PLDs in online learning is complex due to auditory exposure. The pandemic may have increased PLD use not only for online learning but also as a coping mechanism, with studies noting that students used music to mitigate stress during this period [53]. Consequently, both educational and recreational PLD use during the pandemic could collectively heighten the risk for NIHL.

### The impact of COVID-19 on health

There is some evidence suggesting that COVID-19 could have an indirect impact on hearing health. The National Institute for Health and Care Excellence (NICE) [54] has associated long-term COVID-19 with symptoms like tinnitus [55,56], though direct causation has not been substantiated [57]. Establishing a clear cause-effect relationship between COVID-19 and tinnitus is rather problematic, as COVID-19 might also contribute indirectly to the rise in tinnitus cases during the pandemic. For example, stress and anxiety related to the pandemic can also play a role in increasing tinnitus cases. Specifically, stress can lead to the activation of the sympathetic nervous system, prompting the release of hormones like adrenaline and cortisol. This can further lead to physiological changes such as increased heart rate and sweating [58–60]. During the COVID-19 pandemic, chronic stress affected individuals worldwide as strict lockdowns mandated people to stay indoors [61,62], leading to negative impacts on wellbeing and poor mental health [63]. Fear of contracting the virus, lack of treatment, and the high mortality rate associated with the virus further exacerbated psychological distress [64,65]. Extended periods of stress or disrupted lifestyle patterns have been shown to trigger or exacerbate tinnitus [66].

In general, hearing damage due to the use of PLDs has been widely reported. Overall, this effect can vary based on several factors such as the loudness, the proximity of the device to the ear, and the duration of usage. Alternatively, using PLDs responsibly, at moderate volumes and for limited durations, may not necessarily cause immediate damage to hearing. We assume differences in preferred listening levels between music and speech-based content such as online learning. Numerous studies indicate that individuals tend to listen to music at loud levels; this inclination could be attributed to the physiological changes in the brain stimulated by music. Specifically, it has been shown that music has an impact on parts of the brain associated with reward and emotion, prompting the release of dopamine, a neurotransmitter linked to pleasure and reward [67–72]. This neurological influence shapes how individuals perceive and engage with music.

### Limitations and future directions

This study has several notable strengths. First, it provides a national-level perspective on the use of personal listening devices (PLDs) among university students in Jordan, offering insight into auditory health within this specific population. The use of both audiometric testing (including PTA and high-frequency audiometry) and DPOAE measurements adds depth to the investigation of hearing health, enabling a comprehensive evaluation of auditory function. Second, this study addresses the effects of PLD usage in the unique context of the COVID-19 pandemic, when PLD use for online learning became more prevalent. This focus on the pandemic period provides valuable information on the potential long-term effects of PLD use during a time of increased screen and device interaction. Finally, the study considers additional factors, such as tinnitus and hyperacusis, offering a broader view of auditory health than traditional hearing threshold measurements alone.

While the study provides important findings, several limitations must be considered when interpreting the results: Cross-sectional Design: The study’s cross-sectional nature limits the ability to draw conclusions about causality. As data were collected at a single point in time, it is not possible to determine whether PLD usage directly leads to hearing damage or whether pre-existing factors may have influenced both PLD usage and hearing outcomes. Future longitudinal studies would provide stronger evidence of causal relationships.

Self-reported Data: The study relied on self-reported data regarding PLD usage, which may be subject to recall bias or inaccuracies in reporting. Participants may have overestimated or underestimated the duration and volume of their PLD use, which could impact the accuracy of the findings. Objective measures of PLD exposure, such as monitoring actual device usage, could strengthen future research in this area.

Generalizability: The study sample consisted of university students in Jordan, which may limit the generalizability of the findings to other populations. Differences in PLD usage patterns and hearing health between student populations and the general public or other age groups (e.g., adolescents or older adults) may exist, and these results should be interpreted within the context of the specific study group.

Potential Confounding Factors: While this study explored several potential factors related to PLD usage, other variables that may influence hearing health were not fully explored. For instance, the role of genetic predisposition to hearing loss, prior noise exposure (outside of PLD use), and health conditions such as ear infections or medication use could potentially confound the relationship between PLD usage and hearing outcomes.

Lack of Comprehensive Stress and Anxiety Assessment: Given that the COVID-19 pandemic significantly influenced mental health globally, stress and anxiety levels were not comprehensively assessed in this study. Psychological distress could be a contributing factor to tinnitus and other auditory symptoms, and future studies should consider incorporating detailed psychological assessments to explore these potential relationships more thoroughly.

## Conclusion

As online learning becomes increasingly adopted as a standard educational approach, the use of personal listening devices (PLDs) is expected to rise, particularly among students. This shift necessitates a deeper understanding of the potential impact on auditory health, as excessive PLD use may pose risks to hearing. While this study highlights associations between PLD usage and hearing health, it is important to note that causation cannot be conclusively drawn from the data. The observed changes in high-frequency thresholds, though notable, are preliminary and warrant further investigation through longitudinal studies to establish clearer links.

This research underscores the need for developing guidelines and regulations that promote safe listening practices, particularly in educational settings, to protect students’ hearing while balancing the benefits of technology use. Ensuring awareness and adherence to safe listening habits will be crucial in safeguarding auditory health in the context of increased PLD usage for both educational and recreational purposes.

## Supporting information

S1 File Institutional Review Board (IRB) approval document. (PDF)

S2 File Raw data used in the manuscript. (XLSX)

S3 File Proofreading confirmation letter. (DOCX)

## Abbreviations

COVID-19: Coronavirus
THI: Tinnitus Handicap Inventory
HQ: Hyperacusis Questionnaire
kHz: Kilohertz
dB HL: Decibels Hearing Level
DPOAEs: Distortion product otoacoustic emissions
PTA: Puretone audiometry
PLD: Personal listening device
NIHL: Noise induced hearing loss
TTS: Temporary threshold shift
