# Supplementary material for: Personal Listening device (PLD) usage among University Students and their audiometric profile during the shift to online learning post COVID-19
Source: PLoS One. 2025 Mar 25;20(3):e0319665. doi: 10.1371/journal.pone.0319665 (PMC11936156; doi:10.1371/journal.pone.0319665)
Supplement: S1 File — (PDF) [file pone.0319665.s001.pdf]

Ref: / 2023/ 16q Date: 3 / 2023

# To Whom It May Concern

This is to acknowledge that the Institutional Review Board at the University of Jordan (IRB at UJ evaluated the research proposal submitted by Ana'am Alkharabsheh from the School of Rehabilitation Sciences, and which is entitled:

The Effect of Online Learning on the Hearing Characteristics for College Students in Jordan

Decision No. (21-2023): The IRB at UJ approves the conduct of the research referred to above provided that:

- it complies with the guidelines stated in the Declaration of Helsinki and the International Council for Harmonization (ICH);
- the IRB at UJ is notified of any major modifications in the proposed research;
- the research participants, their privacy, and their confidentiality are protected;
- the collected samples and/or data are not used in any unrelated research without prior approval by the IRB at UJ;
- the main researcher informs the IRB at UJ of any harm inflicted on the research participants, whether physical, psychological, or social;
- the IRB at UJ has the right at any time during or after the completion of the research to request the original research material, such as raw data or signed consent forms; and
- the IRB at L^{T}J has the right to suspend its approval in case of major deviations from the proposed research or of any harm inflicted on the research participants.

This approval is valid for one year and must be renewed through submitting a written request by the main researcher(s).

Prof. Faleh Sawair

Chair of the IRB at UJ

Date of the IRB approval application:21/2/2023
