# Supplementary material for: Personal Listening device (PLD) usage among University Students and their audiometric profile during the shift to online learning post COVID-19
Source: PLoS One. 2025 Mar 25;20(3):e0319665. doi: 10.1371/journal.pone.0319665 (PMC11936156; doi:10.1371/journal.pone.0319665)
Supplement: S3 File — (DOCX) [file pone.0319665.s003.docx]

Soha N Garadat

University of Jordan

Amman, Jordan

June 10^th^ , 2024

Dear editor,

This is to confirm that I, the senior author, have proofread the paper titled “**"Impact of Personal Listening Devices Usage on Students’ Hearing Profile During the Shift to Online Learning Post COVID-19**” by Alkharabsheh et al. This included substantial editing in the English language usage, grammar, punctuation, overall style, as well as the manuscript content. I have extensive experience in writing research manuscripts, and I have served as an ad hoc reviewer for the International Journal of Audiology on several occasions.

Sincerely,

Soha N. Garadat, Ph.D.

Department of Hearing and Speech Sciences

University of Jordan Amman, Jordan 11942 garadat@umich.edu s.garadat@ju.edu.jo

[sohagaradat@atsu.edu](mailto:sohagaradat@atsu.edu)
